# Micafungin microevolution in *Candida auris* reveals resistance development without *in vivo* fitness compromise

**DOI:** 10.1080/21505594.2026.2664993

**Published:** 2026-04-29

**Authors:** Flora Bohner, Zora Szilovics, Éva Veres, Csaba Papp, Joshua D. Nosanchuk, Attila Gácser

**Affiliations:** a Division of Infectious Diseases, Department of Medicine, Albert Einstein College of Medicine, Bronx, NY, USA; b Department of Biotechnology and Microbiology, University of Szeged, Szeged, Hungary; c Department of Microbiology and Immunology, Albert Einstein College of Medicine, Bronx, NY, USA; d IKIKK, Competence Centre for Molecular Biology, Bionics and Biotechnology, University of Szeged, Szeged, Hungary; e HCEMM-SZTE Pathogen Fungi Research Group, University of Szeged, Szeged, Hungary; f HUN-REN-SZTE Pathomechanisms of Fungal Infections Research Group, University of Szeged, Szeged, Hungary

**Keywords:** *Candida auris*, antifungal resistance, micafungin resistance, echinocandin resistance, virulence attenuation, systemic mouse model

## Abstract

*Candida auris* is an emerging multidrug-resistant pathogen with high transmissibility in healthcare settings. Although echinocandin resistance in *Candida* is typically associated with fitness loss, we found that micafungin-resistant *C. auris* strains (MICA^evo^) generated from two distinct source isolates (AR0381 and AR0387) via experimental microevolution retained full virulence. Evolved strains developed stable resistance to multiple echinocandins, while AR0387 (B8441), originating from MICA^evo^ strain, also acquired increased azole tolerance. Whole-genome sequencing revealed clinically relevant mutations in *FKS1*, as well as changes in genes involved in ergosterol biosynthesis (*ERG3*), amino acid metabolism, and PKA signaling. Despite *in vitro* sensitivity to cell wall stressors, resistant strains maintained or even enhanced colonization in a murine systemic infection model. Independently evolved strains showed similar antifungal resistance profiles, and although minor differences of pathogenic potential were noted, no consistent virulence attenuation was observed, indicating the reproducibility of phenotype changes. These findings suggest that *C. auris* can acquire echinocandin resistance without compromising pathogenicity, supporting its persistence and spread in clinical settings.

## Introduction

The emerging fungal pathogen *Candida auris* was first described in 2009 [1]. Although the initially characterized isolate (B11220) originated from an ear discharge of an inpatient at a Japanese hospital, *C. auris* strains rapidly emerged globally from geographically distinct locations, resulting in diverse clinical manifestations, including severe bloodstream infections and outbreaks in healthcare centers. Based on location of origin, isolates have been classified into six clades: clade I (South Asia), clade II (East Asia), clade III (Africa), clade IV (South America), clade V (Iran), and the most recently identified clade VI (Singapore) [2,3]. Further examination of these clades revealed distinct clinical associations: while isolates from clade II and V were mainly obtained from ear infections [4], isolates from clade I, III, and IV were predominantly associated with *candidaemia* [5]. It is worth noting that *C. auris* was reclassified based on a single genome analysis as *Candidozyma auris* [6]; however, a working group from the International Society for Human and Animal Mycoses, European Confederation of Medical Mycology, and Fungal Diagnostics Consortium presented a consensus statement for maintaining the use of *Candida auris* [7].

Infections with *C. auris* in a hospital setting are usually associated with alarmingly high mortality rates (23%–67%) [8,9]. Eradication of the pathogen is an issue, as it persists on abiotic surfaces for an extended time, easing its rapid dissemination once introduced into hospital environment [10,11]. The nosocomial spread is further facilitated by the fact that *C. auris* frequently exhibits resistance to commonly used disinfection approaches as well as antifungal drugs [12].

Although resistance patterns vary based on geographical locations, approximately 90% of strains are resistant to fluconazole and subsequently to other triazoles, such as voriconazole [8]. As a result, echinocandins are recommended as the first-line treatment for systemic *C. auris* infections [13]. According to widespread antifungal susceptibility data, micafungin is considered one of the most potent therapeutic and prophylactic agents against *C. auris* [14,15]. However, echinocandin resistance occurs in approximately 5% of isolates, which is higher compared to other clinically relevant *Candida* species (<3%), except for *Candida glabrata*, where in some cases resistance rates exceeding > 5% have been reported [16–18].

Echinocandins are fungicidal drugs that disrupt the cell wall biosynthesis by noncompetitively inhibiting 1,3-β-d-glucan synthase [19]. In *Candida* species, echinocandin resistance generally arises from point mutations in the *FKS1* gene, which encodes the target enzyme. These mutations are typically localized in two highly conserved hot spot regions of the coding sequence [20]. Because of these mutations, resistant strains usually have atypical cell wall compositions, leading to disrupted cell wall homeostasis. This disturbance increases yeast cell sensitivity to perturbation, resulting in increased susceptibility to environmental stress and host immune responses [21]. Due to the fitness cost and the subsequent virulence attenuation, resistance can develop in patients undergoing echinocandin therapy, but the spread of resistant *Candida* strains between patients rarely happens [17]. However, *C. auris* is a notable exception, as echinocandin-resistant isolates can be horizontally transmitted between patients [22,23]. This unique feature suggests that the fitness cost associated with resistance acquisition is significantly distinct in *C. auris* compared to other clinically relevant *Candida* species, permitting more effective human-to-human transmission in addition to environmental spread.

To expand upon our prior study on acquired azole resistance in *C. auris* [24] and study the effects of echinocandin resistance on *C. auris* fitness, we employed our *in vitro* microevolution method to generate micafungin-resistant *C. auris* strains from two distinct drug-susceptible clinical isolates (AR0381 and AR0387). These isolates represent clade II and clade I, respectively. Generally, strains from clade I are isolated from the blood of infected patients and able to acquire antifungal resistance relatively quickly, whereas clade II isolates are more commonly cultured from the skin and less commonly develop resistance. Therefore, the two selected isolates are associated with distinct clinical manifestations and antifungal resistance patterns. Additional control (Ctrl.) strains were also generated by serially passaging the original clinical isolates alongside the evolved strains but without antifungal pressure.

We studied the effects of resistance development by evaluating the abiotic stress tolerance and virulence of these micafungin evolved (MICA^evo^) strains. We found that MICA^evo^ strains developed resistance to all tested echinocandins, and 0387 MICA^evo^ also developed cross-resistance to azoles. The evolved strains were also displayed wild-type levels or, in some cases, higher CFU counts in a systemic murine infection model, suggesting the absence of virulence attenuation. These findings build on the concern for this WHO critical threat pathogen and demonstrate the need for ongoing vigilance for the evolution of resistance during treatment as well as underscore the need for ongoing antifungal drug development.

## Results

### Micafungin microevolution significantly alters antifungal susceptibility of *C. auris* strains

Following micafungin microevolution, antifungal susceptibility testing of the generated strains revealed significant changes. Resistance breakpoints were interpreted according to the tentative CDC guidelines, while dECOFF values (0.125 µg/mL for POS and 1 µg/mL for VOR) were used to differentiate MIC values above the wild-type distribution. Both evolved strains developed stable resistance to MICA, with additional resistance to AND and CAS. While no change of azole susceptibility was identified in the 0381 MICA^evo^ (Table 1A), 0387 MICA^evo^ (Table 1B) had significant increases in MIC values for FLU, POS, and VOR. AMB MIC values were increased in 0387 MICA^evo^, although they stayed below the tentative resistance breakpoint (≥2 µg/ml). While the 0381 Ctrl. strain remained susceptible to every studied antifungal, the 0387 Ctrl. strain displayed increased MIC values for triazoles, with fluconazole even reaching the resistance breakpoint (≥32 µg/ml).

**Table 1.** Antifungal susceptibility testing demonstrating alterations in MICs following the microevolution process.

A														
	MIC (μg/ml)													
	AMB		FLU		POS		VOR		AND		CAS		MICA	
Strains	24 h	48 h	24 h	48 h	24 h	48 h	24 h	48 h	24 h	48 h	24 h	48 h	24 h	48 h
*C. auris* 0381	0.375	0.75	4.5	12	0.0625	0.0625	0.0625	0.0625	0.03125	0.125	0.375	1	0.03125	0.03125
*C. auris* 0381 MICA^evo^	0.375	0.5	2.5	12	0.0625	0.0625	0.0625	0.0625	>16*	>16*	>16*	>16*	>16*	>16*
*C. auris* 0381 Ctr.	0.75	0.75	10	6	0.0625	0.0625	0.25	0.25	0.03125	0. 03125	0.25	0.25	0.03125	0.03125
B														
	MIC (μg/ml)													
	AMB		FLU		POS		VOR		AND		CAS		MICA	
Strains	24 h	48 h	24 h	48 h	24 h	48 h	24 h	48 h	24 h	48 h	24 h	48 h	24 h	48 h
*C. auris* 0387	0.1875	0.5	1.5	8	0.0625	0.0625	0.0625	0.0625	0.078125	0.1875	0.5	0.75	0.03125	0.03125
*C. auris* 0387 MICA^evo^	1	1	>512*	>512*	>32**	>32**	>32**	>32**	4*	4*	>16*	>16*	>16*	>16*
*C. auris* 0387 Ctr.	0.375	0.5	48*	32*	0.53125	0.53125	0.75	1.5	0.3125	0.09375	1	1	0.34375	0.03125

(A-B) MIC values for *C. auris* 0381 (A) and 0387 (B) with the wild-type original isolates, control strains (Ctrl.), and micafungin-evolved strains (MICA^evo^) after 24 and 48 hours of antifungal exposure. Asterisks (*) indicate the emergence of antifungal resistance based on the tentative breakpoints suggested by the CDC. Double asterisks (**) denote potential antifungal resistance according to dECOFF based thresholds. AMB: amphotericin-b; FLU: fluconazole; POS: posaconazole; VOR: voriconazole; AND: anidulafungin; CAS: caspofungin; MICA: micafungin

### Acquired resistance to micafungin slightly alters the growth capability of 0387 MICA^evo^ strains in complex media

**Figure 1.**
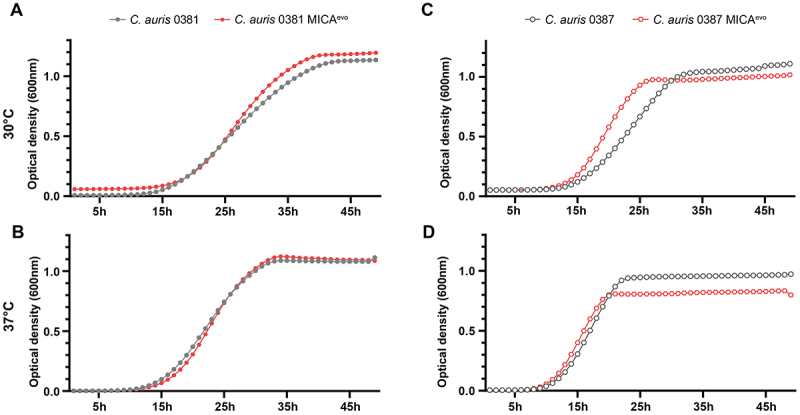
Growth kinetics of micafungin-evolved strains compared to parental isolates in complex media (YPD).

The growth capacity of evolved strains was assessed in YPD media at both 30°C and 37°C (Figure 1). The 0381 MICA^evo^ strain exhibited no significant change in growth compared to its parent isolate (Figure 1(A,B)). In contrast, 0387 MICA^evo^ showed a slight growth advantage at 30°C (Figure 1(C)). This advantage disappears at 37°C, and 0387 MICA^evo^ reaches the plateau phase of the growth capacity at a lower cell concentration than the clinical isolate (Figure 1(D)).

(A, B) Growth curves of the *C. auris* 0381 clinical isolate and micafungin evolved strain (0381 MICA^evo^) at 30°C (A) and 37°C (B). (C, D) Growth curves of the *C. auris* 0387 clinical isolate and micafungin evolved strain (0387 MICA^evo^) at 30°C (C) and 37°C (D). Optical density (600 nm) was measured over 48 hours to assess growth kinetics. Data represents the means of at least 30 data points from three independent experiments.

### Abiotic stress tolerance changes in strain dependent manner upon micafungin microevolution

**Figure 2.**
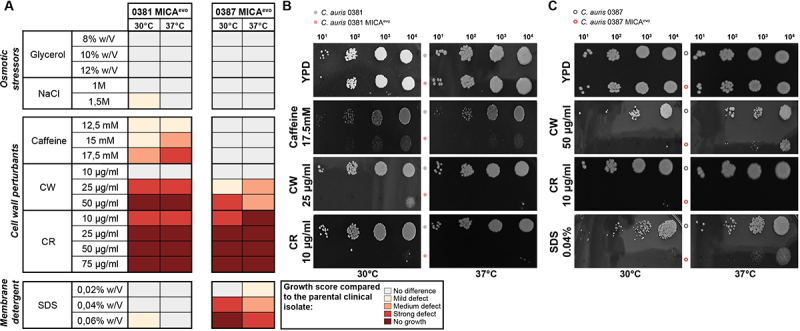
Abiotic stress tolerance of the evolved strains.

Growth capability of the generated strains under stress-inducing circumstances was compared to the clinical isolate using a spot assay. Possible differences of general growth capacity based on control YPD were also considered during the evaluation of the results (Figure 2(A)).

(A) Heat map representing growth responses of the 0381- and 0387-derived micafungin-resistant strains (MICA^evo^) under abiotic stress conditions, including osmotic stressors, cell wall-perturbing agents, and membrane detergents, based on the results of the spotting assay. Growth scores were determined at 30°C and 37°C based on three independent experiments. (B-C) Representative images of the growth of *C. auris* 0381 (B) and 0387 (C) clinical isolates and MICA^evo^ strains on YPD solid media, and media supplemented with stress-inducing agents. CW: calcofluor white, CR: Congo red, SDS: Sodium dodecyl sulfate.

0381 MICA^evo^ showed sensitivity to caffeine-induced stress in a temperature-dependent manner and significantly increased susceptibility to Congo red and high concentrations (25 µg/mL) of calcofluor white (Figure 2(B)).

The 0387 MICA^evo^ strain showed growth comparable to the parental strain in the presence of caffeine, while growth defects at the higher concentrations of calcofluor white (25–50 µg/ml) were identified, as well as high sensitivity to Congo red (Figure 2(C)). We also found reduced growth on plates supplemented with SDS (0.04–0.06% w/V), although this phenotype was less pronounced at 37°C.

Neither of the evolved strains showed changed tolerance to osmotic stressors, such as glycerol or NaCl. Representative images of plates from all studied conditions are presented in Appendix 1. Independently generated control strains were also studied in the presence of cell wall-disturbing agents and exhibited phenotypes comparable to those of the clinical isolates, although slight sensitivity to Congo red was noted (Fig. S1).

### Micafungin resistance changes α-mannan exposure of *C. auris* strains

**Figure 3.**
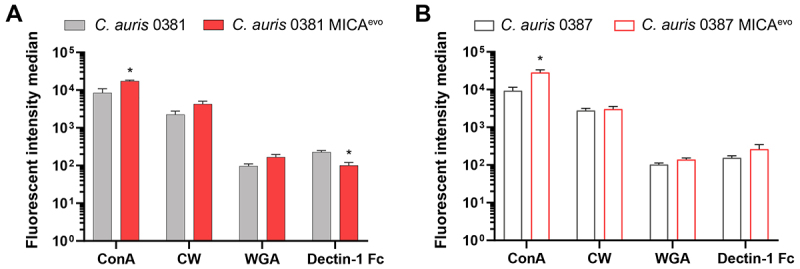
Cell wall alterations of the micafungin-evolved strains.

Fluorescent staining of the cell wall components revealed significantly increased α-mannan (ConA) exposure in both evolved strains compared to the clinical isolates. We registered a two-fold increase of the ConA median fluorescent values of 0381 MICA^evo^ (Figure 3(A)) and nearly a three-fold increase in 0387 MICA^evo^ (Figure 3(B)).

(A, B) Median fluorescence intensity of cell wall components in clinical isolates and evolved strains as determined by flow cytometry. Cells were stained with ConA-FITC (α-mannan), calcofluor white (chitin), WGA-TRITC (chitin oligomers), and AlexaFluor 647-conjugated Dectin-1 Fc antibody (β-glucan). Fluorescence intensity was measured for 3 × 10^4^ cells per strain. Statistical analysis was done by unpaired t-test. Significant differences between clinical isolates and evolved strains are indicated: *, *p* ≤ 0.05. ConA: FITC-conjugated Concavalin-A; CW: calcofluor white; WGA: TRITC-conjugated Wheat Germ Agglutinin; Dectin-1 Fc: Dectin-1: FC (human) Dectin-1 (mouse) antibody.

No significant shift in the chitin or chitin oligomer (WGA) content was found. Interestingly, Dectin-1 Fc staining suggested that β-glucan exposure levels in the evolved strains changed in different directions. In 0381 MICA^evo^, β-glucan exposure significantly decreased, whereas it slightly increased in 0387 MICA^evo^.

### Besides FKS1 mutation microevolved strains acquire several additional genomic changes

Whole-genome sequencing identified multiple nonsynonymous single nucleotide polymorphisms (SNPs) in the 1,3-β-D-glucan coding gene, *FKS1*. In 0381 MICA^evo^ two SNPs were identified (D642Y, R1354S), while 0387 MICA^evo^ harbored a polymorphism in a different position (S639Y) (Table 2).

**Table 2.** Whole-genome sequencing results of the *C. auris* 0381 and 0387 micafungin evolved strains.

*C. auris* 0381 MICA^evo^					
Type of mutation	Gene	*C. albicans* ortholog	*S. cerevisiae* ortholog	Amino Acid Substitution	Function
nonsynonymous SNV	CJI96_0001351 (B9J08_000964) (FKS1)	*GSC1 (FKS1)*	*GSC2 (FKS2)*	D642Y	Putative 1,3-beta-D-glucan synthase, involved in fluconazole resistance
				R1354S	
*C. auris* 0387 MICA^evo^					
Type of mutation	Gene	*C. albicans* ortholog	*S. cerevisiae* ortholog	Amino AcidSubstitution	Function
nonsynonymous SNV	B9J08_002788 (TPK2)	*TPK2*	*TPK2*	R375K	Predicted catalytic subunit of cAMP-dependent protein kinase
stopgain	B9J08_002818	*BCY1*	*BCY1*	S330X	Predicted protein kinase A regulatory subunit
nonsynonymous SNV	B9J08_003737 (ERG3)	*ERG3*	*ERG3*	S243L	Ortholog(s) have C-5 sterol desaturase activity, role in ergosterol biosynthetic process and endoplasmic reticulum lumen localization
nonsynonymous SNV	B9J08_000964	*GSC1 (FKS1)*	*GSC2 (FKS2)*	S639Y	Putative 1,3-beta-D-glucan synthase, involved in fluconazole resistance

Nonsynonymous single nucleotide variants (SNVs) and frameshift variations were identified in *C. auris* 0381 MICA^evo^ and 0387 MICA^evo^ strains that potentially contributed to the development of antifungal resistance. Functions were determined according to CGD [25]. Sequencing was done in three parallels for each strain, and only genomic changes identified across all replicates, relative to the clinical isolate, are listed in this table.

Additional frameshift insertions and deletions in 0381 MICA^evo^ affected two genes, with orthologs involved in Rho signaling (B9J08_000555, B9J08_004831), tryptophan synthesis (B9J08_003349), and methionine homeostasis (B9J08_005307). The latter two suggest that changes of amino acid metabolism may play an indirect role in antifungal resistance acquisition **(Table S1)**.

Alongside the *FKS1* mutation, we identified a presumably loss-of-function (LOF) mutation in *ERG3* (S243L), involved in ergosterol biosynthesis, and in *TPK2* (R375K), encoding the catalytic subunit of the protein kinase A (PKA) pathway. Interestingly, an additional nonsense mutation (S330X) was also found in the regulatory subunit coding gene (*BCY1*) of the same pathway. Another STOP codon (E519X) was identified in *BUD6*, a gene linked to filamentous growth in *Candida albicans*.

### Acquired micafungin resistance significantly alters clearance of C. auris

**Figure 4.**
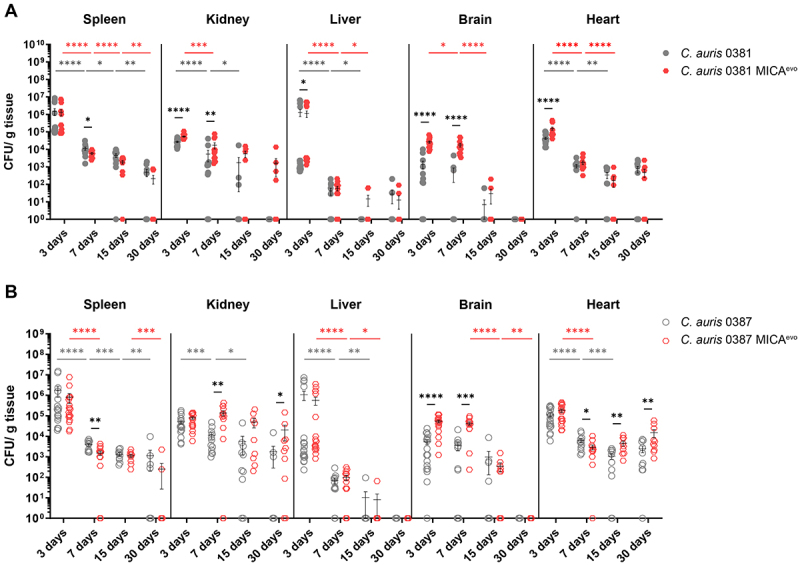
Virulence comparison of the *C. auris* clinical parental isolates and micafungin-evolved strains. The pathogenic potential of the clinical isolates and micafungin-evolved (MICA^evo^) strains was assessed using an *in vivo* systemic mouse infection model. Fungal colonization of kidneys, livers, spleens, brains, and hearts of mice infected with either 0381 clinical isolate or MICA^evo^ (A) was analyzed. The 0387 clinical isolate or MICA^evo^ was similarly used for infection, and tissues from infected mice were analyzed (B). Data points represent CFU values (mean ± SEM) normalized to organ weight registered at 3-, 7-, 15- and 30-days post-infection. Two independent experiments were performed, each with a minimum of 5 animals per group. Mice were infected via the lateral tail vein with 10^6^ fungal cells in 100 µl 1× PBS. Significant differences were determined by Mann–Whitney tests. Statistical significance was defined as follows: *, *p* ≤ 0.05; **, *p* ≤ 0.005; ***, *p* ≤ 0.0005; ****, *p* ≤ 0.0001.

The impact of micafungin resistance development on the virulence of *C. auris* was assessed using a murine model of systemic *candidiasis*. The colonizing capacity of the original clinical isolates was compared to the serially passaged control strains (Fig. S2). At 7 days post-infection, the 0381 clinical isolate exhibited reduced colonization of the brain compared to the generated control strain (0381 Ctrl.) (Fig. S2A). Meanwhile, the 0387 Ctrl. strain displayed lower CFU values in the spleen and heart of the animals compared to the corresponding clinical isolates (Fig. S2B). Three days post-infection, 0381 MICA^evo^ exhibited enhanced colonization of the kidney, brain, and heart compared to the clinical parental isolate (Figure 4(A)). Despite the higher initial burden, over time a steady decrease in CFU levels was observed in most organs, and by day 15, colonization was comparable between the strains. Interestingly, both strains were able to establish a stable cell population in the heart of the animals.

Initially, 3 days after infecting the animals with the 0387 strains, CFU values were comparable between the clinical isolate and 0387 MICA^evo^, except for the brain, where the evolved strain demonstrated significantly higher initial colonization (Figure 4(B)). By day 7, 0387 MICA^evo^ also exhibited enhanced colonization in the kidney, and this phenotype reappeared 30 days after infection. Additionally, 0387 MICA^evo^ infection also led to increased CFU values in the heart on both days, 15 and 30. Similar to the 0381 strains, CFU levels continued to rise between days 15 and 30, suggesting that the organ failed to clear the pathogen. Notably, animals infected with 0387 MICA^evo^ showed neurological symptoms, such as torticollis and erratic movement, starting from 20 days post-infection (Video S1).

### Reproducibility of micafungin-induced phenotypes

To determine whether the observed phenotypic changes were a result of general mechanisms or specific to the individual experiment, the microevolution process was repeated. The individually generated micafungin evolved (0381 MICA^evo^/2, 0387 MICA^evo^/2) strains were further studied.

The 0381 MICA^evo^/2 displayed a resistance profile similar to the original 0381 MICA^evo^ strain, as we detected stable pan-echinocandin resistance (Table 3A). Additionally, the 0387 MICA^evo^/2 strain also showed the same antifungal resistance pattern as the first evolved strain (0387 MICA^evo^), exhibiting drastically increased MIC values for both azoles and echinocandins, rendering it multi-class resistant (Table 3B).

**Table 3.** Results of the antifungal susceptibility test following the second microevolution process.

A														
	MIC (μg/ml)													
	AMB		FLU		POS		VOR		AND		CAS		MICA	
Strains	24 h	48 h	24 h	48 h	24 h	48 h	24 h	48 h	24 h	48 h	24 h	48 h	24 h	48 h
*C. auris* 0381	0.375	0.75	4.5	12	0.0625	0.0625	0.0625	0.0625	0.03125	0.125	0.375	1	0.03125	0.03125
*C. auris* 0381 MICA^evo^/2	0.625	0.75	6	12	0.0625	0.34375	0.25	0.25	>16*	>16*	>16*	>16*	>16*	>16*
B														
	MIC (μg/ml)													
	AMB		FLU		POS		VOR		AND		CAS		MICA	
Strains	24 h	48 h	24 h	48 h	24 h	48 h	24 h	48 h	24 h	48 h	24 h	48 h	24 h	48 h
*C. auris* 0387	0.1875	0.5	1.5	8	0.0625	0.0625	0.0625	0.0625	0.078125	0.1875	0.5	0.75	0.03125	0.03125
*C. auris* 0387 MICA^evo^/2	0.1875	0.5	>512*	>512*	>32**	>32**	>32**	>32**	4*	4*	>16*	>16*	>16*	>16*

(A-B) MIC values for *C. auris* 0381 (A) and 0387 (B) clinical isolates and independently generated micafungin-evolved strains after 24-, and 48-hour antifungal challenge. Asterisks (*) indicate the emergence of antifungal resistance based on the tentative breakpoints suggested by CDC. Double asterisks (**) denote potential antifungal resistance according to dECOFF based thresholds. AMB: amphotericin-b; FLU: fluconazole; POS: posaconazole; VOR: voriconazole; AND: anidulafungin; CAS: caspofungin; MICA: micafungin.

**Figure 5.**
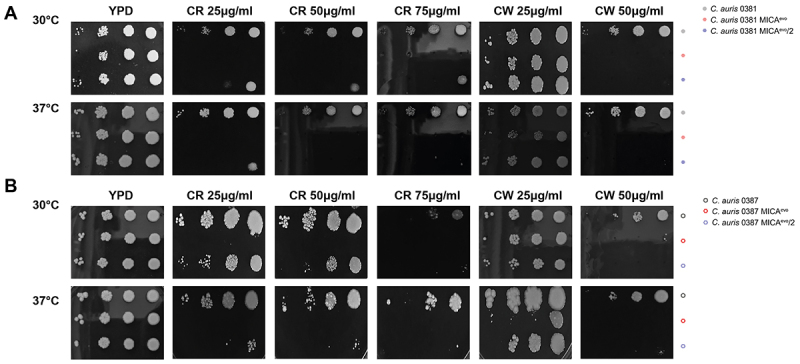
Abiotic stress tolerance of the independently generated *C. auris* strains against cell wall stress inducing agents.

Abiotic stress tolerance was also evaluated in the independently generated strains (Figure 5). Both 0381 micafungin-evolved strains were sensitive to CR; however, this phenotype was slightly less pronounced in the independently generated strain (Figure 5(A)). The 0381 MICA^evo^/2 was more tolerant to low concentrations (25 µg/ml) of CW compared to the original evolved strain (0381 MICA^evo^) at 37°C, though this difference disappeared at higher concentrations (50 µg/ml).

The tolerance of the independently generated micafungin evolved strains to cell wall perturbation was examined by spotting assay. For both 0381 (A) and 0387 (B) strains, MICA^evo^ refers to the original evolved strains, whereas MICA^evo^/2 was used for the independently generated resistant strains.

The 0387 MICA^evo^/2 tolerated CR better than the original evolved strain, especially at 30°C, but the difference leveled off at high concentrations (75 µg/ml) (Figure 5(B)). Similar pattern was identified on plates supplemented with CW, as compared to the original evolved strain. 0387 MICA^evo^/2 grew better on low concentrations (25 µg/ml) at 37°C, but both strains were highly sensitive to 50 µg/ml CR.

### Pathogenic capability of the independently generated strains shows significant difference compared to the originally generated strains

**Figure 6.**
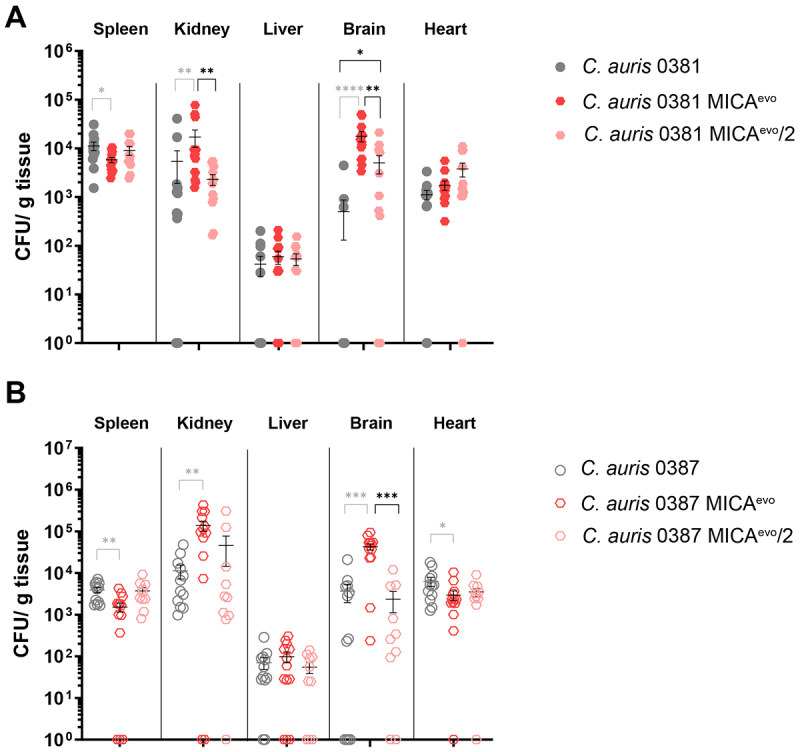
Virulence comparison of the independently generated micafungin-evolved strains.

The virulence of independently generated micafungin evolved strains (MICA^evo^/2) was also assessed at 7 days post-infection to evaluate the consistency of colonization changes induced by the microevolution process (Figure 6). This timepoint was selected as the most pronounced CFU differences between the clinical isolates and the original evolved strains (MICA^evo^) were observed 7 days post-infection. In the animals infected with 0381 MICA^evo^/2, CFU counts were significantly lower in the kidney and brain compared to the original evolved strain (0381 MICA^evo^) (Figure 6(A)). However, brain colonization by 0381 MICA^evo^/2 remained significantly higher when compared to the clinical isolate.

Fungal burdens from animals infected with the 0381 clinical isolates and micafungin-evolved strains (A) and 0387 clinical isolates and micafungin-evolved strains (B) were compared to evaluate the consistency of the virulence changes caused by micafungin selection. CFU levels were determined at 7 days post-infection, and data values (mean ± SEM) were standardized to the weight of each organ. Two independent experiments were conducted, with at least five animals per group. Mice were infected via the lateral tail vein with 10^6^ fungal cells in 100 µl 1× PBS. Significant differences were determined using Mann−Whitney tests (grayed-out significance levels were discussed previously), and significance was defined at: *p ≤ 0.05; **p ≤ 0.005; ***p ≤ 0.0005.

Similarly, CFU levels in the brain were decreased in animals infected with the 0387 MICA^evo^/2 compared with those infected with the original 0387 MICA^evo^ strain, whereas in other organs, CFU levels were comparable to the clinical isolate; thus, no attenuation of virulence was noted (Figure 6(B)).

## Discussion

The high frequency of azole and amphotericin-B resistance in *C. auris* increases the need for the use of echinocandins, such as micafungin, as both a prophylactic agent [26] and a first-line treatment option for systemic infections [13]. However, and perhaps consequently, clinical isolates with acquired echinocandin resistance, capable of horizontal transmission, have also emerged in healthcare settings [9,27,28]. *In vitro* microevolution is a widely utilized and powerful tool for understanding the development of antifungal resistance [20,29–31]. Although our group [24] and others have previously generated and characterized resistant *C. auris* strains, these studies primarily focused on polyenes, azoles, and caspofungin [32–34]. Therefore, in this study, we applied a microevolution approach to generate multiple micafungin-resistant strains using two clinical isolates (AR0381 from clade II and AR0387 from clade I) that originated from patients with distinct clinical manifestations. We then examined both the impact and reproducibility of micafungin resistance development in these two isolates.

Antifungal susceptibility testing indicated that all generated strains acquired resistance to all tested echinocandins (AND, CAS, MICA). Additionally, 0387 MICA^evo^ also showed resistance to all tested azoles (FLU, POS, VOR), indicating the acquisition of cross-resistance in the absence of specific selective pressure. To confirm the reproducibility of this phenotype, independently evolved strains (0381 MICA^evo^/2 and 0387 MICA^evo^/2) were also analyzed. Similar MIC values between the biological parallels suggest the universal nature of the altered resistance phenotype, albeit the genetic alterations for individual generated strains may vary.

The genomic backgrounds of the MICA^evo^ strains were analyzed by whole-genome sequencing. In the 0381 MICA^evo^ strain, two amino acid substitutions (D642Y and R1354S) were identified in the *FKS1* (B9J08_000964) gene. A combined mutation of this locus has previously been reported in clinical isolates from patients undergoing micafungin treatment [35]. Both D642Y (HS1) and R1354S (HS2) mutations have separately been previously confirmed to confer echinocandin resistance in *C. auris*. However, susceptibility testing of isolates carrying D642Y demonstrated that they do not always reach the resistance threshold [36]. In *Candida parapsilosis*, substitutions outside the canonical hot-spot regions can confer echinocandin tolerance that may facilitate the development of resistance [37]. The combined occurrence of these mutations in both clinical isolates and the 0381 MICA^evo^ suggests that stepwise adaptation under echinocandin exposure, similar to *C. parapsilosis*, may occur in *C. auris*, which could be further explored in the future. Similarly, the S639Y substitution identified in the *FKS1* coding region of the 0387 MICA^evo^ has been previously described in both murine models [38] and in patients receiving echinocandin therapy, underscoring the clinical relevance of these alterations [27,35]. Although identified *FKS1* modifications have been observed in clinical isolates from patients undergoing micafungin treatment, targeted genetic validation will be required to confirm the individual and combined contributions of these mutations to the resistant phenotype.

Beyond *FKS1* modifications, an additional non-synonymous substitution (S243L) was identified in the *ERG3* (B9J08_003737) gene of the 0387 MICA^evo^ strain. Mutations in the gene encoding the C5 sterol desaturase enzyme (*ERG3*) are frequently associated with azole resistance in several *Candida* species [20], including *C. auris* [39]. Furthermore, mutations in genes involved in ergosterol biosynthesis can emerge following echinocandin treatment in *C. auris* and related species, such as *C. lusitaniae* [40–42]. Recently, LOF mutations in *ERG3* have also been reported to contribute to echinocandin resistance acquisition in *C. glabrata*, as altered membrane sterol composition may influence ligand binding ability of the Fks enzyme. Based on these observations, as well as other studies, Carolus *et al.* proposed a potential epistatic interplay between the *ERG3* and *FKS* variations [43]. Although the exact nature of this cross-talk remains unclear, the frequent occurrence of these combined genome alterations has been hypothesized to drive MDR development. While increasing evidence supports the clinical relevance of this interaction, thorough experimental validation is still required to understand this complex association. Therefore, future studies with targeted genetic validation of the S243L mutation and sterol composition analysis of the 0387 MICA^evo^ strain should potentially broaden our understanding of this observation.

To assess the impact of microevolution, control strains were generated by serially culturing the parental clinical isolates (AR0381 and AR0387) without drug exposure. In these cell cultures the absence of antifungal selection led to extremely high cell densities. Even with periodic YPD medium replacement and limited oxygenation, the high biomass potentially created an environment with persistent nutrient depletion and hypoxia. Both starvation and oxygen deficiency are known to induce broad transcription changes in *Candida* species, including alterations in ergosterol biosynthesis [44,45]. Based on this, we hypothesize that the FLU resistance observed in 0387 Ctrl., along with the increased MIC values to other antifungals, may be linked to this adaptation process. Consequently, since the transcriptional changes occurred independently of resistance development, these strains cannot be considered true controls in the microevolution experiment.

Abiotic stress resistance of the generated strains revealed altered tolerance to cell-wall perturbing agents at varying levels. Notably, 0381 MICA^evo^ exhibited sensitivity to the presence of caffeine at 15, and 17.5 mM, while this phenotype was absent in 0387 MICA^evo^, as it showed comparable growth to the clinical isolate. 0387 MICA^evo^ also displayed slightly altered growth capability in complex media in temperature-dependent manner. Using whole-genome sequencing in 0387 MICA^evo^, we identified an R375K substitution in B9J08_002788 and an S330X nonsense mutation in B9J08_002818. In *C. albicans* these genes, *TPK2* and *BCY1*, encode key components of the cAMP-dependent protein kinase A (PKA) pathway [46]. In our previous work, we identified alterations in this multifunctional pathway in similarly generated azole-resistant strains exhibiting high caffeine tolerance [24]. Since caffeine is known to target the Target of Rapamycin (TOR) pathway, our finding further suggests a potential, yet unexplored, interaction between these regulatory pathways in *C. auris* [47]. While based on early genome analysis, Chatterjee et al. proposed the possible involvement of the PKA pathway in the antifungal resistance, and Zamith-Miranda et al. reported the increased relative abundance of *TPK2* in resistant isolates, no variations in *BCY1* have been documented in clinical *C. auris* isolates thus far [48,49]. Despite its uncertain role, components of the *C. auris* PKA pathway were analyzed in the past, with results indicating that the cAMP/PKA pathway plays an important role in the adaptation to environmental nutrition availability as well as both drug resistance and virulence [50].

Cell wall composition strongly determines fungal virulence as it serves as a primary interface between the pathogen and host immune system. The overall structure of this cell organelle is highly conserved [51]. Similar to other *Candida* species, the cell wall of *C. auris* consists of two distinguishable layers. The inner layer contains a skeletal chitin layer (β-1,4-linked N-acetyl glucosamine homopolymer) and a less tightly packed glucan network. The outer layer is built from mannosylated glycoproteins [51,52]. Although the increased presence of chitin was not significant in either evolved strain, in *C. albicans* elevated chitin levels can confer echinocandin resistance; therefore, we can presume the same might be true for our generated strains [53]. Notably, cell wall composition of *C. auris* possesses unique immune-evasive features, as the most immunogen β-(1,3)-glucan layer is more extensively masked by mannans, which can shield the pathogen from immune recognition [52].

Consistent with the known role of *FKS1* mutations in cell wall biosynthesis, micafungin-evolved strains exhibited increased sensitivity to CW and CR, both agents known to perturb cell wall integrity [54,55]. Interestingly, cell-wall analysis revealed an overall expansion of α-mannan in both micafungin-evolved strains. However, while 0381 MICA^evo^ displayed reduced β-glucan (Dectin-1 Fc) levels with the slight increase in chitin (as determined by CW quantification) as a compensatory mechanism, 0387 MICA^evo^ exhibited a trend toward increased β-glucan concentrations. These results suggest that the identified *FKS1* mutations in the evolved strains differentially affected the kinetics of β-(1,3)-glucan synthase (Fks1) [56], indicating distinct resistance mechanisms at the molecular level. These findings support the hypothesis that *C. auris* cell wall architecture is less dependent on β-(1,3)-glucans compared to *C. albicans* [33] and further emphasize the differences in adaptive processes between distinct clades. Progressive microevolution in *C. haemulonii* has been associated with cell-wall rearrangement, increased abiotic stress sensitivity, and attenuated virulence [57]. This is in line with other *Candida* species, as the development of echinocandin resistance is generally linked to fitness costs and virulence attenuation, which limits the spread of resistant strains in healthcare settings [21,53,55]. However, *C. auris* presents a notable exception since nosocomial outbreaks of resistant strains are well documented [23,58]. This indicates that the effect of the fitness loss associated with resistance acquisition in *C. auris* is less severe compared to other *Candida* species. *C. auris* infections generally yield a high fungal burden in the heart and kidney in neutropenic mice model [59–61]. In line with this, according to clinical case studies, patients who succumb to systemic *C. auris* infections frequently develop renal- and/or cardiac failure, indicating the central role of these organs in determining virulence [62,63].

To study the impact of resistance development on virulence, we used a systemic mouse infection model and monitored organ colonization at different intervals until 30 days post-infection. Early after infection (3 and 7 days), the 0381 MICA^evo^ strain was able to colonize the host at levels comparable to or, in the case of kidney and brain, exceeding those of the clinical isolate. By 15 and 30 days post-infection, with the clearance of the organs, the difference between the strains diminished. Notably, CFU levels indicated the establishment of a stable fungal population in the heart following infection with either 0381 or 0387 MICA^evo^ strains, which could potentially explain the high frequency of cardiac complications reported in patients with *C. auris* infections. Additionally, 0387 MICA^evo^ exhibited increased kidney colonization at 30 days and enhanced brain colonization at early time points (3 and 7 days). Although survival was not affected, mice infected with 0387 MICA^evo^ developed severe neurological symptoms (e.g. erratic movement, torticollis, and tail spinning) approximately 20 days post-infection, indicating central nervous system (CNS) impairment [61,64]. Clinical data suggest that *C. auris* can cause CNS infections, with some patients even displaying altered mental states and unsteady movements [63,65]. Because systematic neurological scoring, histopathology, and dedicated CNS-focused analyses were not performed, this observation should be interpreted cautiously. Nevertheless, central nervous system infection by *C. auris* has been reported in clinical settings, and neurological manifestations have been described in some cases, and these findings will be studied in detail in the future.

Innate immune cells detect fungal cell wall components through pattern recognition receptors (PRRs), with β-glucan sensed primarily by Dectin-1, whereas mannans are recognized by multiple receptors, including C-type lectin receptors such as Dectin-2, the mannose receptor (MR), and DC SIGN and can also engage TLR2 and TLR4, which together promotes phagocytosis and inflammatory signaling [66]. In our micafungin-evolved strains, the increased mannan exposure could explain the lack of virulence attenuation, although decreased β-glucan exposure and possible masking were only observed in the 0381 MICA^evo^ strain [66,67]. More broadly, the suggestion that cell wall remodeling is responsible for the lack of measurable virulence changes as a compensatory mechanism opens a way to exploit this vulnerability as a future therapeutic option, for example, by using combination strategies that target mannosylation pathways or other non-glucan cell wall processes to destabilize adaptation processes and inhibit indirect β-glucan masking.

To determine whether the observed virulence changes were specific to our strains or represented a more general effect, pathogenicity of the subsequently generated strains 0381 MICA^evo^/2 and 0387 MICA^evo^/2 was also evaluated. Compared with the initial resistant MICA^evo^ strains, 0381 MICA^evo^/2 exhibited reduced colonization in the kidney and brain at day 7 post-infection, while a similar decrease in CFU counts was observed for 0387 MICA^evo^/2 in the brain. However, despite the resistance acquisition, neither set of the evolved strains displayed attenuated virulence relative to the corresponding clinical isolates. These results indicate that *C. auris* strains can potentially retain their ability to colonize the host at levels comparable to the nonresistant isolates, even after acquiring micafungin, or even multidrug resistance. Consistent with our findings, Kiyohara *et al*. reported that experimentally generated *FKS1* mutant *C. auris* strains originating from a distinct set of clinical isolates also maintained their colonizing ability in systemic infection models using immunosuppressed mice, further supporting the apparent uniformity of this unique feature [68].

Collectively, our findings demonstrate that *C. auris* isolates can acquire micafungin resistance without the attenuation of *in vivo* fitness in our systemic infection model, in contrast to observations reported in other *Candida* species. In addition, some resistant strains may even exhibit enhanced organ colonization and induce neurological symptoms, an observation that warrants focused follow-up experiments incorporating histopathology and host immune reaction studies. The potential for the emergence of multidrug resistance, as observed in 0387 MICA^evo^, underscores the urgent need for stringent infection control and reevaluation of micafungin prophylaxis. Our findings provide experimental evidence that certain *C. auris* strains can develop echinocandin resistance while retaining their colonization capacity, thereby paving the way for persistence in healthcare environments and reinforcing the urgency of utilizing alternative antifungal strategies and therapeutic development, such as targeted combination strategies to destabilize the remodeled cell wall.

## Materials and methods

### Strains and culturing conditions

For each experiment, fungal strains were cultured in 5 ml YPD liquid media (1% D-glucose; 1% peptone; 0.5% yeast extract) supplemented with 1% Penicillin-Streptomycin and incubated at 30°C with 200 rpm shaking overnight. A 150 µl aliquot of cells was subcultured for an additional 16 hours, collected by centrifugation (2.4 *g*, 5 min), and washed three times with 1× PBS.

Clinical isolates of *C. auris* were maintained on YPD agar plates, while micafungin-evolved strains were grown on YPD agar supplemented with 8 µg/mL micafungin (cat. no: SML2268; Sigma-Aldrich). *C. auris* strains used in this study were purchased from CDC & FDA Antibiotic Resistance Isolate Bank (Atlanta, GA, United States). Between experiments, strains were kept at 4°C and stored long-term at −80°C in YPD liquid media with 20% glycerol.

### In vitro microevolution process

The *in vitro* microevolution process was performed as established previously by Papp et al. [69] with adjustment to antifungal concentrations. Briefly, the optical density of clinical isolates (0381 and 0387) was adjusted to 0.1 (OD_600 nm_) in YPD medium. Initial micafungin concentrations were set at half the MIC values for each isolate (AR0381: 0.0625 µg/mL (https://wwwn.cdc.gov/ARIsolateBank/Panel/IsolateDetail?IsolateID=381&PanelID=2); AR0387: 0.25 µg/mL (https://wwwn.cdc.gov/ARIsolateBank/Panel/IsolateDetail?IsolateID=387&PanelID=2). Following 24 hours of incubation, cell cultures were centrifuged daily and resuspended in fresh YPD media supplemented with the previously used drug concentration. After 96 hours of drug exposure, optical density was adjusted again to 0.1 (OD_600 nm_) in YPD media, and cells were incubated with the previously used drug concentration for 10 hours. After the incubation, equal volume of micafungin was added to each culture to double the previous drug concentration. The following day (after 14 h incubation), the increased drug concentration was maintained as previously established. By repeating this cycle, fungal cells were exposed to stepwise increases in micafungin concentrations every 96 hours until reaching a final concentration of 16 µg/mL. Adapted strains were subsequently passaged for 10 days in drug-free YPD medium and plated on solid YPD agar. Resistant strains are referred to as evolved (MICA^evo^) strains. Control strains (Ctrl.) were cultured under identical conditions but without micafungin exposure. For this, the optical density of the clinical isolates was adjusted to 0.1 (OD_600 nm_) in YPD medium, and the cells were then incubated with shaking at 150 rpm at 30°C for 24 hours. After incubation, cell cultures were centrifuged, and cell pellets were suspended in fresh YPD daily for 96 hours. On day 5, optical density was again adjusted to 0.1 (OD_600 nm_), and the same cycle was repeated until the microevolved strains reached the maximum antifungal concentration.

### Antifungal susceptibility testing

Antifungal susceptibility is assessed based on the M27 Ed. 4^th^ standard protocol [70]. The range of the tested antifungal concentrations was modified for *C. auris*. Susceptibility was determined for each strain by using the following antifungals: amphotericin B (AMB) [cat. no: A4888; Sigma-Aldrich], fluconazole (FLU) [cat. no: PHR1160; Sigma-Aldrich], posaconazole (POS) [cat. no: 32,103; Sigma-Aldrich], voriconazole (VOR) [cat. no: 32,483; Sigma-Aldrich], anidulafungin (AND) [cat. no: SML2288; Sigma-Aldrich], caspofungin (CAS) [cat. no: SML0425; Sigma-Aldrich], and micafungin (MICA) [cat. no: SML2268; Sigma-Aldrich]. Resistance was defined following CDC guidelines [71]. MIC breakpoints for posaconazole and voriconazole were extended using dECOFF (epidemiological cutoff) methods [14].

Microdilution tests were executed in RPMI 1640 media (cat. nr: 12-115F; Lonza) supplemented with MOPS (cat. nr: M3183; Sigma-Aldrich). Stock solutions of each antifungal were prepared according to the manufacturer’s instructions. The most concentrated drug solutions were prepared from the stocks in RPMI 1640-MOPS, and two-fold serial dilutions were performed to obtain lower drug concentrations. The 2× drug concentrations were dispensed into rows 1 to 10 of 96 well microdilution plates in 100 μl volume. To bring drug dilutions to final concentration 100 μl of 3 × 10^3^ fungal cells were seeded in the corresponding wells. Row 11 was used as drug-free cellular control, while row 12 was used as medium-only sterility control. *Candida paraspilosis* ATCC 22,019 and *Candida krusei* ATCC 6258 strains were used as quality controls. MIC values were determined after 24 and 48 hours of incubation at 35°C. For echinocandins, a 50% decrease in growth and for other antifungals, a 90% decrease in growth were considered as inhibition. Two biological replicates were conducted in three technical parallels per antifungal for each strain.

### Growth in complex media

Fungal cells were prepared as described above. Cell concentration was adjusted (10^4^ cells/200 μl) in liquid YPD in 96-well microtiter plates. Plates were then incubated in a spectrophotometer at either 30°C or 37°C with OD_600 nm_ measured hourly over 48 hours. Growth curves were generated by the means of hourly OD values. Each strain was tested in eleven technical replicates across three independent experiments.

### Examination of the abiotic stress tolerance

Spotting assays were used to assess abiotic stress tolerance on osmotic (glycerol, NaCl) and cell wall (caffeine, calcofluor white, Congo red) stressors and a membrane detergent (SDS) supplemented YPD plates. Stressors were chosen to represent possible conditions that fungal cells encounter upon host infection. Serial dilutions of washed fungal cells were prepared to set 2 × 10^6^, 2 × 10^5^, 2 × 10^4^, and 2 × 10^3^ cells/ml concentrations. Five μl of the dilutions were spotted onto stressor-supplemented or standard (YPD) agar plates to reach the final concentrations of 10^4^, 10^3^, 10^2^, and 10^1^. For each agent the following concentrations were applied: 8%, 10%, and 12% (wt/vol) glycerol; 1 M and 1.5 M NaCl; 12.5 mM, 15 mM, and 17.5 mM caffeine; 10 μg/ml, 25 μg/ml, and 50 μg/ml calcofluor white; 10 μg/ml, 25 μg/ml, 50 μg/ml, and 75 μg/ml Congo red; 0.02% (wt/vol), 0.04% (wt/vol), and 0.06% (wt/vol) SDS. Plates were incubated at 30°C and 37°C, and the growth capability of evolved strains was compared to the initial clinical isolate, considering possible growth rate differences that may be evident on standard YPD media. Results were evaluated using a subjective scoring system with previously defined categories [72]. Spotting experiments were performed with 3 biologic replicates.

### Fluorescent cell wall staining

Fungal cells were prepared as above and suspended in 200 µL 1× PBS +1% BSA (cat. nr: A7906; Sigma-Aldrich), followed by incubation at 30°C with continuous rotation for 30 minutes. Cells were washed twice in 1× PBS and suspended in 100 µL of 1× PBS +1% BSA containing fluorescent dyes at the following concentrations: 8 μl 2.5 mg/ml ConA-FITC (Concanavalin A), 1 μl 1 mg/ml CW (Calcofluor white), 1 μl 1 mg/ml WGA-TRITC (Wheat Germ Agglutinin). Samples were stained for 30 minutes at 30°C with rotation, washed three times in 1× PBS, and suspended in 100 µL 1× PBS.

For β-glucan staining, fungal cells were suspended in 100 μl FCB (flow cytometry block) solution, containing 0.5% BSA and 2 mM NaN_3_ diluted in 1× PBS. Cells were then pelleted (0.8 g for 2 mins) and washed three times with 200 μl ice-cold FCW (flow cytometry washing) solution (0.5% BSA in 1× PBS) and incubated with 1 µg/mL Fc (human): Dectin-1 (mouse) antibody in 100 µL FCB for 30 minutes. Cells were then washed three times (200 µL FCW) and incubated with Alexa Fluor 647-conjugated anti-human IgG Fc antibody (1:200 ratio) in FCB for 45 minutes on ice. After three final washes with ice-cold FCW, cells were suspended in 50 µL of 1× PBS and kept on ice until analysis. Median fluorescent intensity of 10^4^ cells was measured by Amnis FlowSight flow cytometer, and data were analyzed with IDEAS 6.2 software. For each strain, at least three individual experiments were performed.

### Murine in vivo studies

The pathogenicity of the clinical isolates and subsequently generated strains was determined using a murine systemic *candidiasis* model. Female BALB/c mice (8–10 weeks, 20–21 g weight) were provided by Charles River Laboratories, purchased through the official local supplier AnimaLab Hungary Ltd. Animals were infected intravenously with 100 µL of fungal suspension containing 10^6^ fungal cells. For each experiment, negative control animals were injected with 100 µL of 1× PBS solution. At indicated timepoints, mice were euthanized with gradual CO_2_ exposure, and liver, spleen, kidney, brain, and heart were collected and homogenized in 1 ml 1× PBS solution. 100 µl of homogenate was plated on YPD agar (supplemented with 1% Penicillin-Streptomycin) and incubated at 30°C for 48 hours. Organ colonization was quantified as CFU/g tissue. Five mice were used per group in at least two independent experiments. Sample size calculation for the *in vivo* experiments was conducted by PS Power and Sample Size Calculation software (Version 3.1.6, October 2018). Data were analyzed using GraphPad Prism 7.00 software. Significant differences were determined using the Mann−Whitney test, with *p* < 0.05 considered statistically significant.

### DNA extraction for whole genome sequencing

Washed fungal cultures were suspended in 500 µL lysis buffer (1× TE, 0.1 M NaCl, 2% Triton X100, 1% SDS). Glass beads (0.25–0.5 mm) were added to disrupt cells by vortexing for 3 minutes. 270 µl of ammonium acetate (5 M) was added to disrupted cells. Samples were incubated at 65°C for 5 minutes and then cooled on ice for 5 minutes, followed by the addition of 500 µL chloroform-isoamyl alcohol (24:1). After centrifugation (16.2 *g*, 10 min), the upper aqueous phase was transferred to new tubes, and isopropanol was added at a 1:1 ratio. Samples were incubated at −20°C for at least 45 minutes and centrifuged (16.2 *g*, 10 min). After discarding the supernatant, 500 µl of 70% EtOH was added to each sample. Ethanol was removed (16.2 g, 5 min), and the pellet was air-dried at room temperature before being dissolved in 25 µL of 1× TE buffer. DNA samples were stored at −20°C until use.

### Genome sequence analysis

Whole genome sequencing (WGS) was performed by Novogene Co., Ltd., with each strain sequenced in three independent replicates. Mutations were considered valid only if they were present in all replicates of the evolved strains and absent in all replicates of the initial isolates.

Quality check and quantification of the DNA were assessed by agarose gel electrophoresis and Qubit® 3.0 fluorometer. NEBNext® DNA Library Prep Kit was used for the construction of the DNA library, and library quality was confirmed using the Qubit® 2.0 fluorometer and Agilent® 2100 bioanalyzer. Sequencing was performed on the Illumina® platform with paired-end reads of 150 bp (PE150).

Raw sequencing data were converted to sequence reads using CASAVA software (v1.8), and quality control was performed. Cleaned reads were aligned to reference genomes using BWA (v0.7.8-r455), and duplicates were removed using SAMTools (v0.1.19-44428 cd). For *C. auris* 0381 clinical isolate and 0381 MICA^evo^ strain ASM301371v2 (GCA_003013715.2) genome, it was used as a reference, while *C. auris* 0387 and 0387 MICA^evo^ strains were mapped to Cand_auris_B8441_v2 (GCA_002759435.2). Single nucleotide variations (SNPs) and InDels (≤50 bp) were analyzed by SAMTools (0.1.19-44428 cd). Structural variations (SVs > 50 bp) were detected with BreakDancer (v1.4.4), while copy number variants (CNVs) were identified using CNVnator (v0.3). Alterations were annotated using ANNOVAR (2015Mar22). The functional effects of amino acid substitutions were predicted using PROVEAN (v1.1), with the default deleterious variant threshold score set at −2.5. Supplementary table S1 contains mutations not discussed in this manuscript. Gene functions were obtained from CDG [25] and SGD [73].

## Supplementary Material

FigS2_Bohner_et_al_in_vivo_wt_ctrl_comparation.tiff

FigS1_Bohner_et_al_spotting_control_plates.jpg

TableS1_Bohner_et_al_additional_seq.docx

## Data Availability

The data supporting the finding of the study, the completed ARRIVE checklist and supplementary video (Video S1) are openly available in Figshare, under https://doi.org/10.6084/m9.figshare.30359086.v2 [74]. Raw sequencing data for clinical isolates 0381 and 0387 is available under BioProject accession numbers (SAMN05379608 and SAMN05379624) with assembly accession numbers (GCA_003013715.2 and GCA_002759435.2). SRA data for the generated strains can be accessed under the following reference number: PRJNA1285280. Biosample reference of the 0381 MICA^evo^ can be accessed using SAMN49760343−SAMN49760345 (SRR34334838, SRR34334839, SRR34334840) reference number. Biosample reference of the 0387 MICA^evo^ can be accessed using SAMN49760346−SAMN49760348 (SRR34334835, SRR34334836, SRR34334837) reference number.

## Statistical tests

Data evaluation was performed by GraphPad Prism 7.00 software. For statistical analysis, the unpaired t-test and Mann–Whitney test were used. Statistical significance was determined using the following *p*-values: *, *p* ≤ 0.05; **, *p* ≤ 0.005; ***, *p* ≤ 0.0005; ****, *p* ≤ 0.0001.
