# Supplementary figures and images for: Micafungin microevolution in Candida auris reveals resistance development without in vivo fitness compromise
Source: Virulence. 2026 Apr 29;17(1):2664993. doi: 10.1080/21505594.2026.2664993 (PMC13134414; doi:10.1080/21505594.2026.2664993)

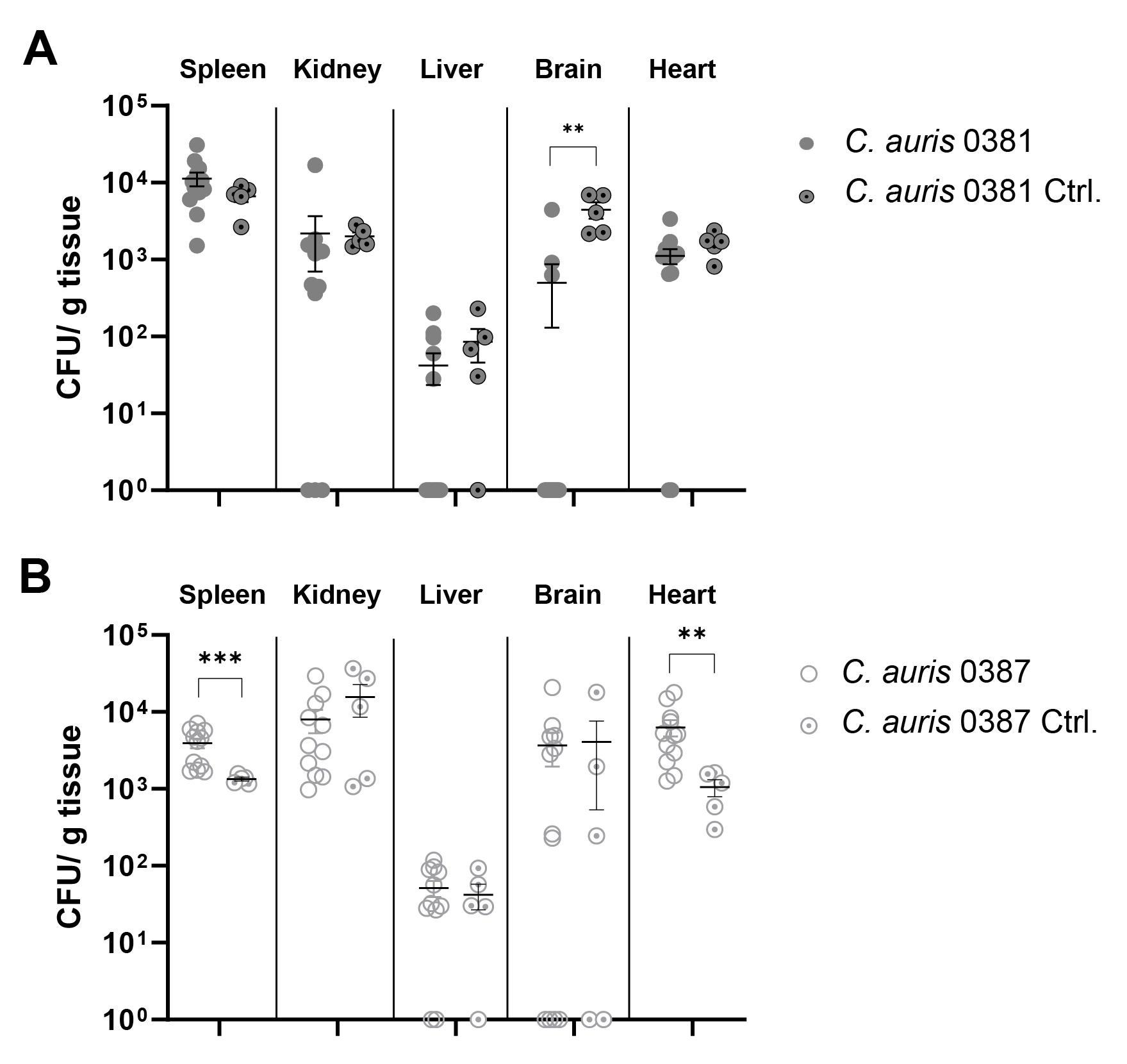

Supplement: FigS2_Bohner_et_al_in_vivo_wt_ctrl_comparation.tiff [file KVIR_A_2664993_SM8636.tiff]

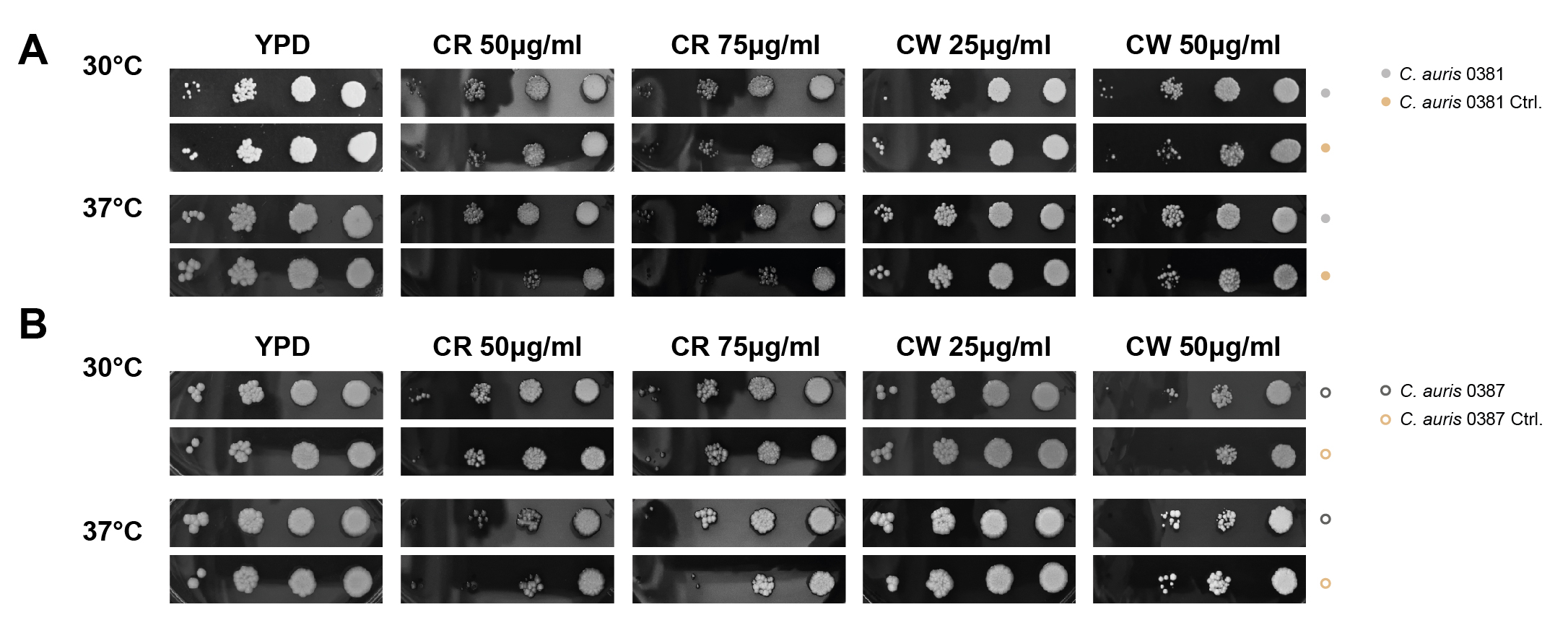

Supplement: FigS1_Bohner_et_al_spotting_control_plates.jpg [file KVIR_A_2664993_SM8635.jpg]
