# Supplementary material for: Micafungin microevolution in Candida auris reveals resistance development without in vivo fitness compromise
Source: Virulence. 2026 Apr 29;17(1):2664993. doi: 10.1080/21505594.2026.2664993 (PMC13134414; doi:10.1080/21505594.2026.2664993)
Supplement: TableS1_Bohner_et_al_additional_seq.docx [file KVIR_A_2664993_SM8634.docx]

|  | | | | ***C. auris* 0381 MICA^evo^** | | |  |
| --- | --- | --- | --- | --- | --- | --- | --- |
| **Type of mutation** | **Gene** | ***C. albicans* ortholog** | ***S. cerevisiae* ortholog** | | **Amino Acid Substitution** | **Function** | |
| **nonsynonymous SNV** | CJI96_0003689 (B9J08_004899) | orf19.1430 | - | | M59I | Protein of unknown function | |
| **frameshift insertion** | CJI96_0001886 (B9J08_003349) | *TRP5* | *TRP5* | |  | Ortholog(s) have tryptophan synthase activity and role in cleistothecium development, tryptophan biosynthetic process | |
| **frameshift insertion** | CJI96_0003540 (B9J08_000555) | C6_02090C_A | *PXL1* | |  | Ortholog(s) have Rho GDP-dissociation inhibitor activity and role in maintenance of cell polarity, regulation of Rho protein signal transduction | |
| **frameshift insertion** | CJI96_0004094 (B9J08_005307) | *MET16* | *MET16* | |  | Ortholog(s) have phosphoadenylyl-sulfate reductase (thioredoxin) activity and role in sulfate assimilation, sulfate assimilation, phosphoadenylyl sulfate reduction by phosphoadenylyl-sulfate reductase (thioredoxin); | |
| **frameshift deletion** | CJI96_0001295 (B9J08_001347) | *SAC7* | *SAC7* | |  | Ortholog(s) have GTPase activator activity; | |
| **stoploss** | CJI96_0004322 (B9J08_004831) | *AAF1* |  | |  | Possible regulatory protein; possible adhesin-like; Glu-rich domain; production in S. cerevisiae increases endothelial cell adherence and flocculence; flow model biofilm, alkaline or caspofungin induced | |

|  | | | | ***C. auris* 0387 MICA^evo^** | | |  |
| --- | --- | --- | --- | --- | --- | --- | --- |
| **Type of mutation** | **Gene** | ***C. albicans* ortholog** | ***S.cerevisiae* ortholog** | | **Amino Acid Substitution** | **Function** | |
| **nonsynonymous SNV** | B9J08_000053 | *TOM20* | *TOM20* | | L108F | Ortholog(s) have mitochondrion targeting sequence binding, protein transmembrane transporter activity; | |
| **stopgain** | B9J08_004233 | *BUD6* | *BUD6* | | E519X | Ortholog(s) have actin binding, cytoskeletal regulatory protein binding, enzyme activator activity | |
| **frameshift insertion (*renders the orf to be in frame)** | B9J08_001051 | *C1_01540W_A* | *RUP1* | |  | Ortholog(s) have role in positive regulation of protein autoubiquitination, protein deubiquitination | |
| **stoploss** | B9J08_002272 | *C3_03560W_A* | *ICP55* | |  | Ortholog(s) have aminopeptidase activity, role in protein processing, protein stabilization and extrinsic component of mitochondrial inner membrane, mitochondrion, nucleus localization | |
